# Waterbody loss due to urban expansion of large Chinese cities in last three decades

**DOI:** 10.1038/s41598-022-22286-x

**Published:** 2022-10-19

**Authors:** Wu Xiao, Wenqi Chen, Wenze Yue, Jingxuan Mu, Jianpeng Xu

**Affiliations:** 1 Department of Land Management, Zhejiang University, Hangzhou, 310058 China; 2 Land Academy for National Development, Zhejiang University, Hangzhou, China

**Keywords:** Urban ecology, Environmental impact, Sustainability

## Abstract

Urban waterbodies are one of the most pertinent issues involved in multiple aspects of Sustainable Development Goals (SDGs). However, waterbodies in large Chinese cities are highly vulnerable to urban-land expansion, which is mostly due to economic development, population growth, and rural–urban migration. In this work, we selected 159 Chinese cities of over one million in population to investigate the encroachment on waterbodies due to rapid urbanization from 1990 to 2018. Overall, 20.6% of natural waterbody area was lost during this period to urban expansion, and this fraction varied from city to city which was related to waterbody abundance. With the acceleration of urbanization, waterbody occupation is becoming more serious (*P* < 0.01). However, in all cities, this encroachment has eased since 2010, which justifies the effective implementation of national-scale policies to conserve urban waterbodies. Meanwhile, gains have occurred during urbanization, in addition to the loss of waterbodies. Especially, cities lacking waterbody placed a greater emphasis on ecological factors, whose urban waterbody areas showed an increasing trend. In the future, ecological resources, including waterbody, should be considered in urban planning to provide reasonable protection to waterbodies in the quest for urban sustainability.

## Introduction

Waterbodies are vital for cities^1^ by providing drinking water, transportation, and cultural activities such as leisure tourism^2,3^. The sixth objective of the Sustainable Development Goals (SDGs) is sustainable water conservation and management. In particular, urban waterbodies maintain the stability of local ecosystems and provide a wide range of ecosystem services, including biodiversity conservation, groundwater supplementation, and climate adjustment^4–7^. Furthermore, urban waterbodies are also part of networks that help regulate rainfall and contribute significantly to the city’s capacity to accommodate and recover rapidly from extreme weather and other natural disasters.

Over the past decades, economic development and population growth in China fuelled by rural–urban migration have driven the demand for urban land^8^. From 2001 to 2018, the growth rate of China’s built-up land has been the highest in the world, contributing to nearly half of the total rise globally^9^. Complex human activities based on urban expansion are constantly changing the planet, leading to deteriorated resources, frequent climate extremes, and severe perturbations of biochemical cycles^10–13^. China’s rapid urbanization is characterized by extensive expansion based on a high rate of consumption of natural resources^14^. Impervious surfaces have replaced vast tracts of ecological areas, leading to drastic ecological degradation^15^. Studies have shown that changes in land use and associated pressures have reduced the biodiversity of global land surfaces by up to 58%^16^. Massive green space, water, and other natural habitats have deteriorated and/or been converted to different uses, which now poses a significant challenge to urban ecological security^17^. As a result, urban growth in China is now subject to the dual constraints of ecological space and environment.

Widespread urban expansion is encroaching on waterbodies all over the world^18,19^, particularly in the large cities of Asia due to the recent rapid urbanization of developing countries^20–22^. On one hand, the agglomerated population and the limited land of large cities make waterbody occupation more serious in large cities. On the other hand, urban planning in large cities is generally more systematic and comprehensive. According to the literature, waterbodies in large Chinese cities have been severely reduced and/or transformed into urban construction land^23–25^. Most previous studies explored spatial–temporal changes in waterbodies and made specific suggestions for future urban planning on a regional basis^22,26–28^. In China, extensive wetlands disappeared during urbanization, totalling 2883 km^2^ in 1990–2010, and this situation got worse^29,30^. What features of losses are present in cities with various water-abundance? And how is the temporal change of waterbody loss at different urbanization levels in China?

To deal with its rapid development, China is exploring a new path of sustainable urban planning^29^. In 2007, waterbody conservation was reinforced in Chinese urban planning, and the Administrative Measures of the Urban Blue Line were issued to restrict development activities on urban waterbodies. In 2013, China proposed the sponge city program, emphasizing adopting of the low impact development concept to achieve harmonious development of resources and environment. Reports have also discussed artificial water and water protection as cities strive for liveable space. Has a turning point been reached in people’s concept of waterbody protection? This paper aims to quantitatively assess how urban expansion affects waterbodies in Chinese cities of over one million. Towards this end, we analyzed the dynamics of urban boundaries and waterbodies over different periods from 1990 to 2018 and explored whether and how waterbodies are affected by urban expansion.

## Results

### Unbalanced distribution of waterbodies and waterbody loss

**Figure 1:**
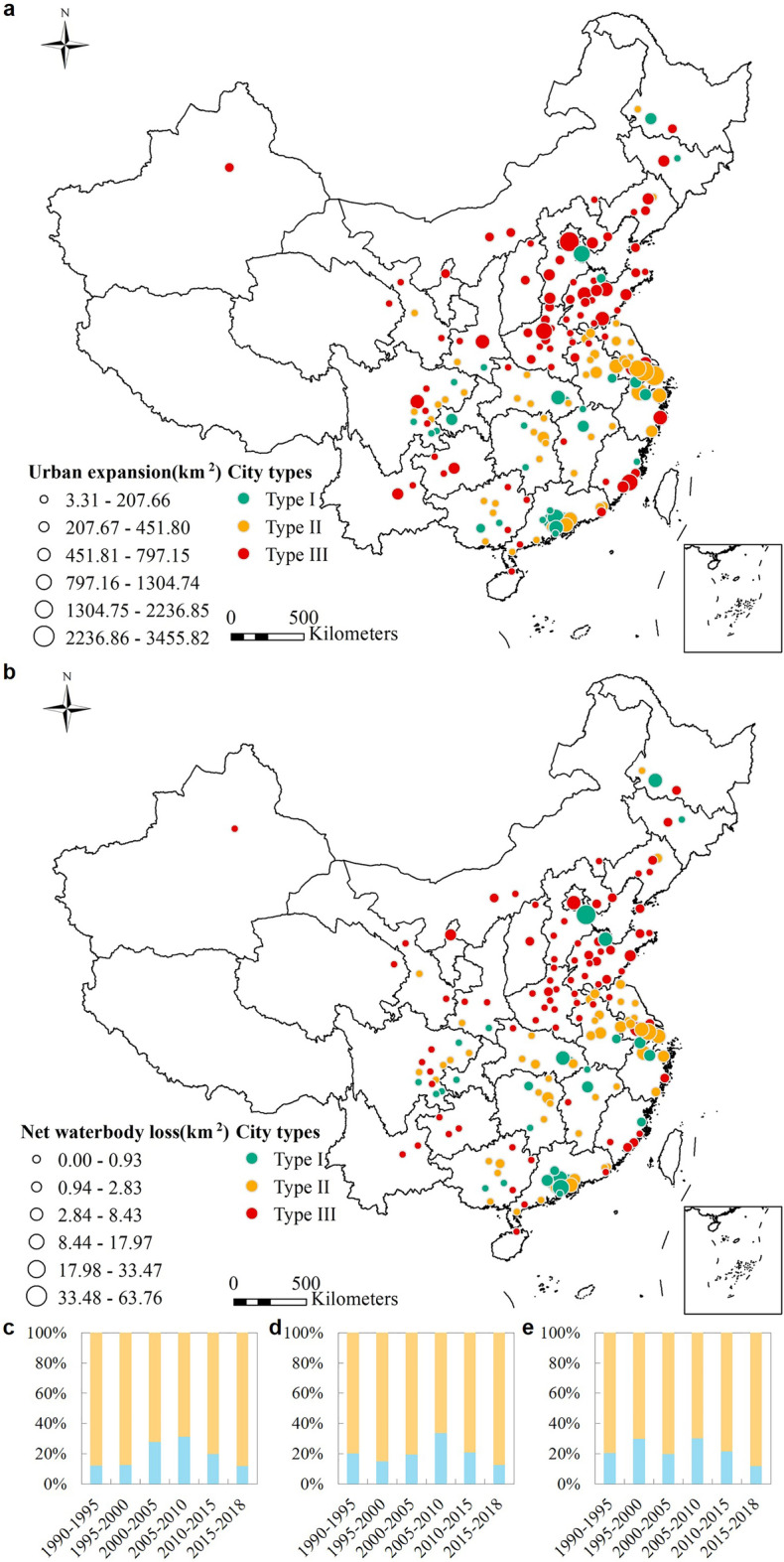
Waterbody loss in urban expansion: (**a**) Spatial distribution of urban expansion in different types of cities. (**b**) Spatial distribution of waterbody loss in different types of cities. (**c**)–(**e**) Waterbody loss (WL) and waterbody preservation (WP) rate as a function of time in areas of urban expansion in cities of Type I–III, respectively. The map was generated by ESRI ArcGIS 10.2 software available at ESRI website (https://www.esri.com/en-us/arcgis/products/arcgis-platform/overview). And the administrative boundary shapefile is available at RESDC website (https://www.resdc.cn/).

Only about 2% of the urban area is devoted to waterbodies in cities on average out of 2090.89 km^2^ (Fig. 1a,b). Zhaoqing city, Guangdong Province, has the highest fraction of waterbodies at 10.3%, and 16 cities devote less than 0.1% of their area to waterbodies (Fig. S4a). This reveals the significant regional differences in waterbody development in highly urbanized cities. From 1990 to 2018, these cities lost 464.3 km^2^ of waterbodies, which is 20.6% of the original area devoted to waterbodies (Table S1). According to the waterbody abundance in cities, we classified the cities into three categories from abundant to deficient, including Type-I, -II and -III, and more detail in Material. This waterbody loss is mainly concentrated in Type-I and -II cities and accounts for 245.1 and 143.9 km^2^ of waterbody loss. Type-III cities devote the least amount of land area to waterbodies (only 75.3 km^2^ in total). In general, a city with abundant waterbodies will encroach more on them as a result of its expansion. Furthermore, the occupation of waterbodies increased continuously from 1990 to 2010, following which it decreased drastically in urban areas. The period 2005–2010 saw the greatest loss of 183.7 km^2^, of which over half occurred in Type-I cities.

Although the three types of cities differ significantly in terms of waterbody loss, these differences are less significant in proportion to the original waterbody. Significant areas of waterbodies were preserved despite urban expansion, but the waterbody loss rate increased before 2010, particularly for Type-I and -II cities (Fig. 1b–d). The peak 5-year waterbody loss in all three types of cities was from 2005 to 2010 (31.2%, 33.5%, and 30.3% for Type-I, -II, and -III cities, respectively). In contrast, the 5-year waterbody loss of Type-I cities was only 12.1% from 1990 to 1995. A similar waterbody-loss trend occurred in Type-II cities. The rate of waterbody loss in Type-III cities was much greater from 1990 to 2000 and fluctuated (Fig. 1e). After 2010, however, the rate of waterbody loss for all three types of cities declined significantly.

### Waterbody encroachment due to urban expansion

**Figure 2:**
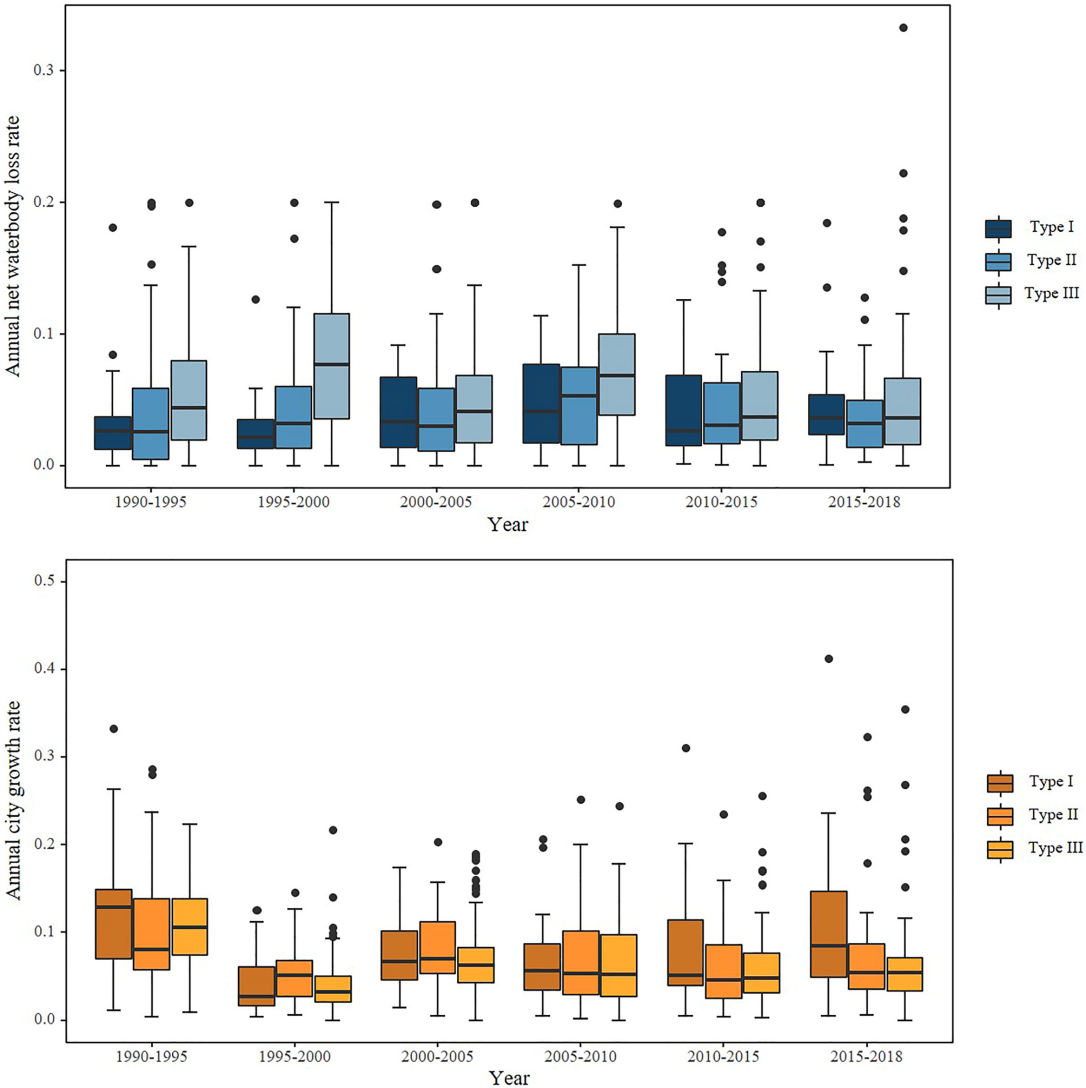
Annual net waterbody loss rate and city growth rate as a function of time for cities of Type I–III.

In Chinese cities of over one million in population, the urban area increased from 13,194.8 km^2^ to 81,622.1 km^2^ in 1990–2018, for an average expansion of 23.9 km^2^ per year overall (Figs. 1a, and S2). The overall rate of urban growth slowed steadily during this time, decreasing from 11.59% in 1990–1995 to 7.43% in 2015–2018 (Fig. 2). In addition, the growth rate was essentially the same for cities with different waterbody abundance. Cities that underwent significant urban expansion are concentrated mainly in the four urban agglomerations with high rates of economic development. These cities were sizable in 1990, and still underwent large-scale expansion; the expansion area reached 1000 km^2^ over the past 30 years. However, many cities in China achieved a high rate of urban expansion on a smaller city size (i.e. urban areas of fewer than 50 km^2^ in 1990). For example, the built-up area of Putian City in Fujian Province increased nearly 67-fold from 5.92 km^2^ in 1990 to almost 400 km^2^ in 2018 (Table S2).

The annual average net waterbody loss (ANWL) rate in every city varies significantly over time (Fig. 2). The ANWL for each type of city is 3.9%, 4.2%, and 6.1%, respectively, and peaked at 6.0% in 2005–2010. Type-III cities underwent the largest variation in ANWL, mainly because of a lack of original waterbody. Mild waterbody loss can lead to dramatic changes. The ANWL rate increased from 1990 to 2010, following which it decreased and then stabilized.

**Figure 3:**
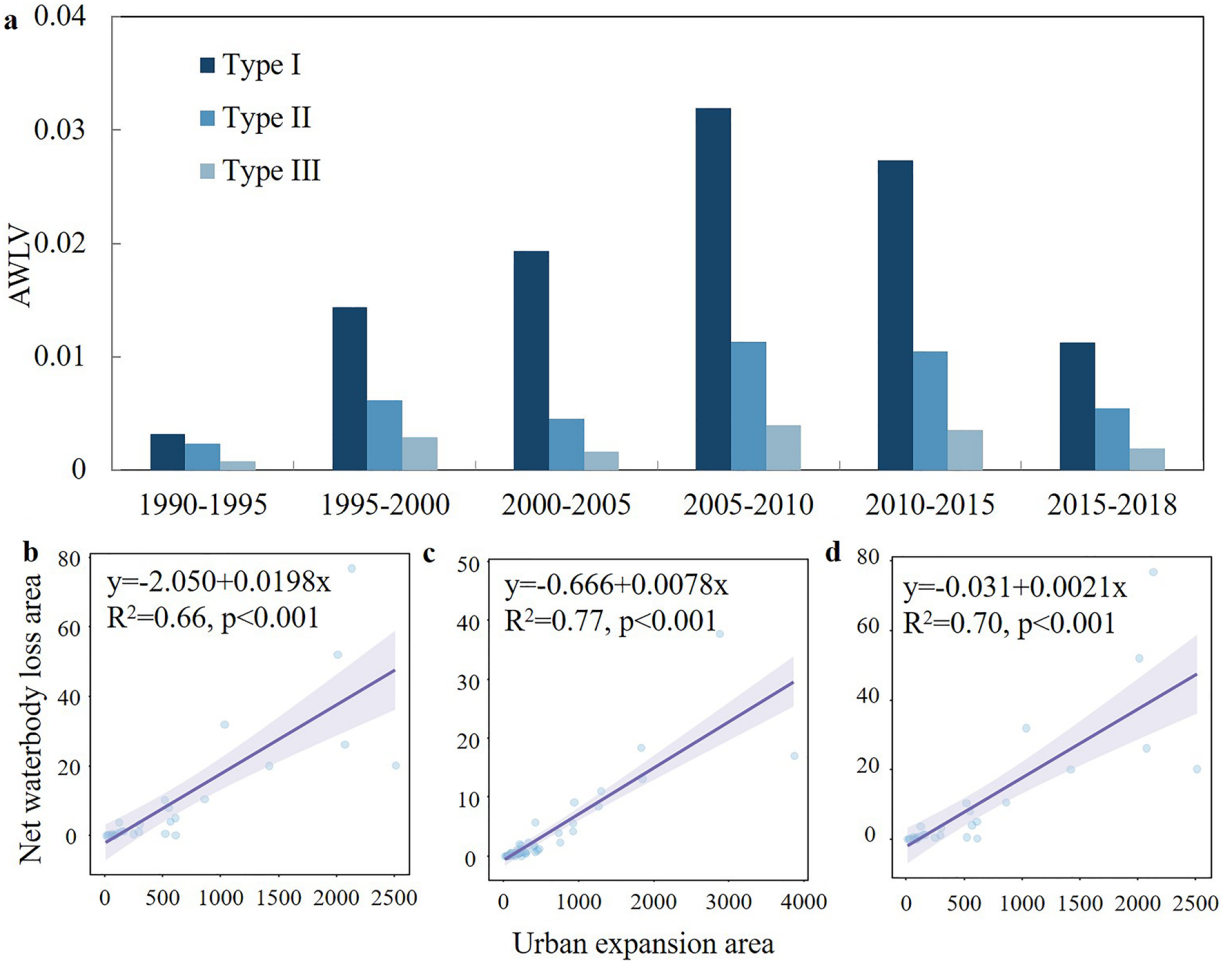
Rate of waterbody loss as a function of urban expansion area. (**a**) Overall AWLV. (**b**)–(**d**) Spearman correlation analysis of waterbody loss as a function of city expansion area for cities of Type I–III, respectively.

Variations in waterbody coverage may be affected by the rate of urban expansion. We illustrated the actual waterbody variation by using the waterbody loss per unit urban expansion area, which is independent of city size and expansion rate. A Spearman correlation analysis showed that waterbody loss correlates strongly with the rate of urban expansion (statistical significance *P*-value < 0.01) (see Fig. 3b–d). For Type-I cities, the average net waterbody loss velocity of urban expansion (AWLV) was only 0.003 in 1990–1995, which means 0.003 km^2^ of waterbodies were occupied when the city expanded by 1 km^2^ (Fig. 3a). However, the AWLV increased ninefold from 2005 to 2010, which represents a huge waterbody loss. Since 2015, the AWLV has decreased to 0.01, which is the 2000–2005 level. The AWLV for the three types of cities increased first and then decreased and tended to be less for cities with rich waterbody at the outset. Wuhan, known in China as the city of a thousand lakes, has abundant waterbody resources, covering 139.3 km^2^ and accounting for 8.4% of the urban area. However, the waterbody area in Wuhan decreased from 1990 to 2018, with 28 km^2^ of waterbody area being occupied due to urban expansion. The fraction of waterbody loss for the period 2005–2015 even exceeded the fraction of waterbody retained (Table S1).

In contrast with Type-I cities, the AWLV for Type-II and -III cities decreased from 2000 to 2005. In general, the indices for Type-II and -III cities are much lower than that for Type-I cities, which peaked at 0.011 and 0.003, respectively. The phenomenon of water encroachment rarely occurred during urban expansion in water-deficient cities due to the small size of waterbodies (Fig. 6(3)). The velocity of waterbody loss for the three types of cities increased first and then decreased. Meanwhile, the rate of waterbody loss decreased significantly for cities with an initial lack of waterbodies. Water-rich cities thus may have neglected to protect their waterbodies from rapid urban expansion.

### Overall change in urban waterbody area

Up to 38.4% of waterbody area was encroached upon from 1995 to 2000, which represents a significant loss of waterbody area in water-deficient cities (Fig. 1c). Although waterbody area was significantly encroached upon due to urban expansion, it was also augmented in other ways. From 1990 to 2018, 407.3 km^2^ of waterbody area was added in urban expansion areas, of which 154.4, 129.1, and 123.8 km^2^ were added in Type I–III cities, respectively. Type-III cities have a greater rate of annual average net waterbody gain (ANWG), with an average of 36.6%, because these cities lacked waterbodies at the outset (i.e. in 1990). Type-I and -II cities experienced a slightly greater rate of urban expansion (16.6% and 25.9%, respectively).

**Figure 4:**
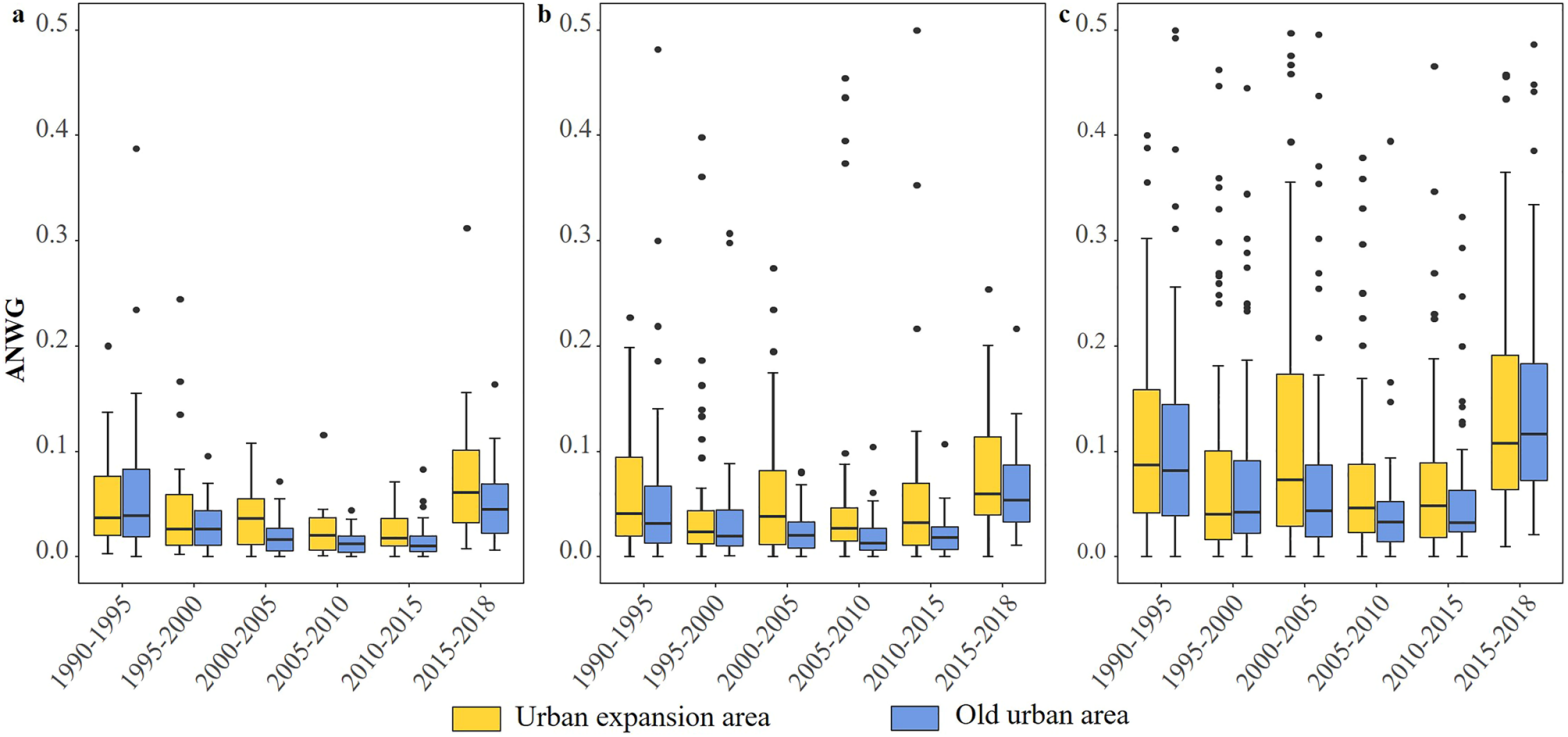
Box diagram showing ANWG index in urban expansion areas and old urban areas for (**a**)–(**c**) cities of Type I-III, respectively. For visual, some ANWG values above 0.5 in Type-III cities are not shown in the figure.

In addition to the urban expansion area, we also found that waterbody area was added in old urban areas (Fig. 4). In old urban areas, the ANWG index decreased gently from 1990 to 2010 and then increased from 2010 to 2018. In Type-I and -II cities, the ANWG of old urban areas is less than that of urban expansion areas, although this is not the case in Type-III cities, where waterbody replenishment in old urban areas outstripped even the urban expansion areas. This demonstrates not only a large gain in waterbody area during urban expansion but also the importance of protecting and developing waterbodies in old urban areas.

**Figure 5:**
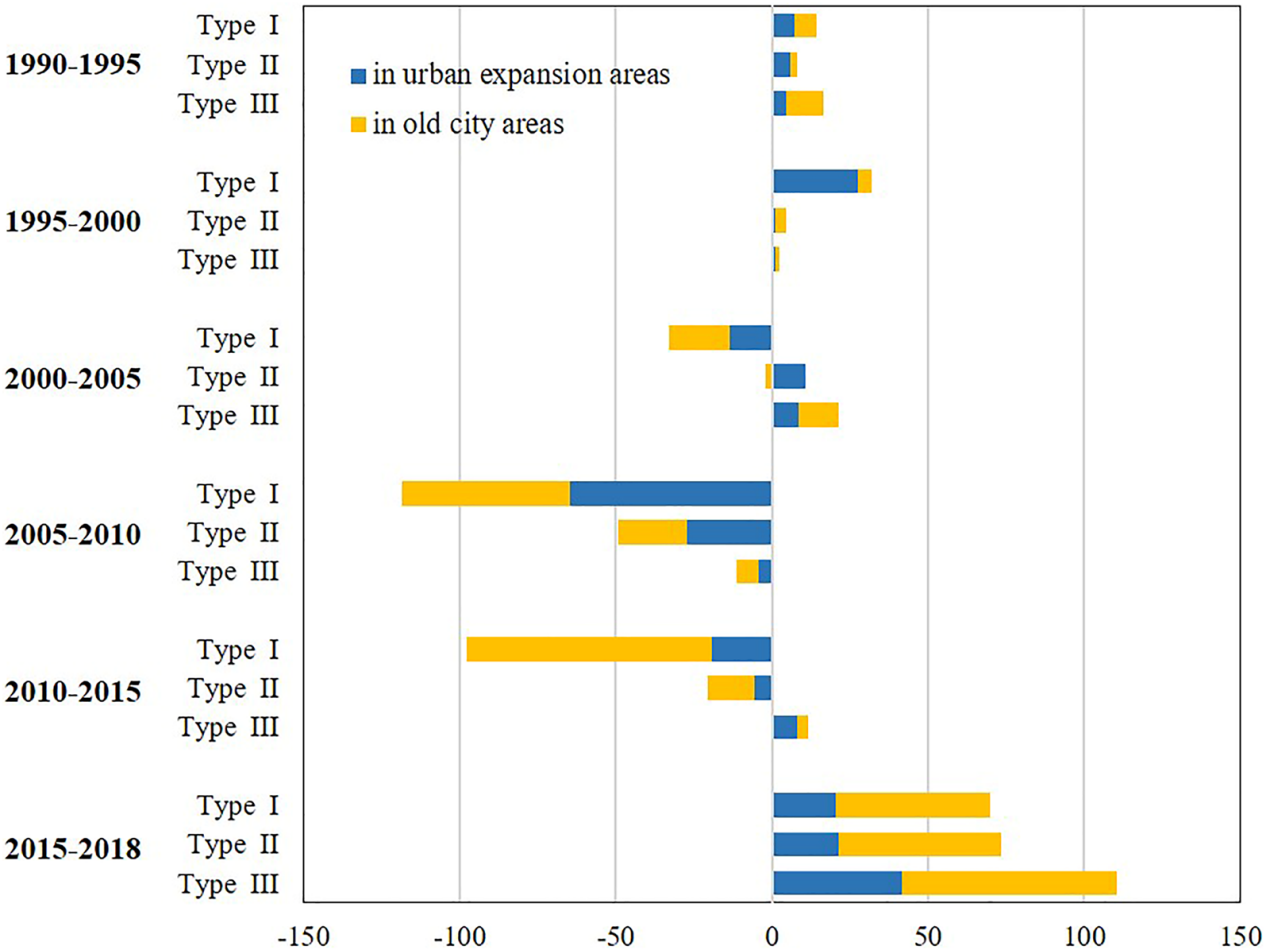
Urban waterbody variation for each period for the three city types.

The total waterbody changes in urban areas can be obtained by summing the urban expansion area and the old urban area at each stage. The result is that waterbodies in all cities mainly decreased from 2005 to 2015 and then increased from 2015 to 2018 (Fig. 5). The waterbody area decreased from 2000 to 2015 in Type-I cities, with gross waterbody loss reaching as high as 118.5 km^2^ within city boundaries from 2005 to 2010. From 1990 to 2018, gross waterbody loss reached 133.5 km^2^. In Type-II cities, the overall waterbody area remained constant from 1990 to 2005, with an occasional increase, until suffering a significant loss in 2005–2015. As a typical Type-II city, Changsha has relatively little waterbody with a waterbody area that accounts for 2% of its urban area. However, it suffers from negligible waterbody encroachment. The preserved waterbody area is 22.7 km^2^, and the loss was only 3.8 km^2^ due to urban expansion from 1990 to 2018, with the most serious loss occurring in 2005–2010. From 1990 to 2018, the waterbody gain increased for Type-II cities, exceeding the loss by about 25.3 km^2^ on average.

While in Type III cities, waterbody gain thus exceeds waterbody loss. Waterbody gain occurred in various periods, except for 2005–2010. In other words, although a net waterbody loss occurred overall, waterbody gain occurred in other areas of the cities. Zhengzhou is a typical water-deficient city, where the urban waterbody occupies only nine-thousandths of the city’s area. Therefore, waterbodies are rarely involved in urban expansion in Zhengzhou, and only 1.3 km^2^ of waterbodies were encroached upon from 1990 to 2018. In contrast, up to 12.3 km^2^ of new waterbodies were developed in the urban expansion. Thus, significant waterbody gain occurred in Zhengzhou from 2010 to 2018, far exceeding the waterbody loss.

## Discussion

Massive waterbodies are occupied and converted to other land-use types in the process of urban expansion in China. Both Mao’s and our study provide support for this conclusion^30^. Mao et al. (2018) described the severity of wetland loss to urbanization by estimating wetland changes in different geographic regions. Our study concentrated on the temporal variation in the waterbody loss to urban expansion among cities with different water-abundance in rapidly urbanizing regions, reflecting the characteristics and concept of waterbody protection in China's urban expansion. In general, waterbody loss correlates positively with waterbody abundance. In addition, the waterbody distribution is not balanced between the city types, with Type-I and -II cities and their more abundant waterbodies losing significantly more waterbody due to urban expansion. Existing research focuses on the protection and management of mostly water-deficient regions^31^. However, serious problems currently exist regarding waterbodies, requiring more attention to waterbodies all over the world. More than half of the world’s rivers or streams currently are non-perennial^32^, and the connectivity of rivers is also weakening, producing a risk of flow interruption^33^. Lakes are also shrinking worldwide^34^. Although these changes are strongly affected by natural factors, human activities cannot be ignored, which may affect the future spatial distribution of waterbodies^35,36^.

Waterbody loss is related to the scale of urban expansion. The larger urban expansion scale, the more likely waterbody encroachment. For instance, severe waterbody encroachment occurred in several urban agglomerations in China with rapid social and economic development, mainly in the Beijing-Tianjin-Hebei Region, the Yangtze River Delta, the Wuhan metropolitan area, and the Pearl River Delta, although these areas are not with the same extent of urban waterbodies in China (Fig. 1). Other investigators reported similar results for nationwide wetland loss, which includes lakes, rivers, and reservoirs^30^. The primary reason for these tragedies is the policies and regulations introduced recently to promote urban development. Urban infrastructure and other urban development projects support local urbanization but require land for construction, which leads to encroachment on waterbodies^37^. In addition, the urban population has increased rapidly in China, with the fraction of urban population increasing from 17.9% to 52.6% from 1978 to 2012^38^, increasing the demand for construction land. Given the visible benefits of land finance, the boom in economic development zones and real estate has led to lake filling and reckless housing construction. Finally, the increase in impervious surface area caused by urban expansion also negatively affects waterbodies^39^.

Built adjacent to the water is a crucial component of urban development since surface waterbodies can support the regional economy and social co-development. The uneven distribution of resources, including waterbody may lead to uneven regional economic growth in China. Fast-growing cities tend to have abundant waterbody, while water-scarce regions typically have a low level of development (Fig. 2). It is difficult to portray water justice issues through constant waterbodies because of the vast and uneven distribution of waterbodies. However, the study is focused on cities of over a million people, representing issues of surface waterbody encroachment and retention under various waterbody-abundance in the concept of environmental justice and fairness. Water-deficient cities protect the original waterbody significantly during urbanization and even improve the environment by constructing landscapes about water, which can reduce the gap with water-rich cities. China has also made some progress in resolving the issue of the unequal allocation of water resources between the north and the south by creating water conservation initiatives like the South-North Water Transfer. Meanwhile, there is no discernible difference between old urban areas and urban expansion areas in the ANWG (Fig. 4), and there is little evidence that old urban areas have a superior ecological landscape due to economic growth. Waterbody gain, such as artificial lake and park pond contribution, can improve the surrounding environment. And it did not show significant spatial differentiation in the waterbody gain, although socio-economic distinctions in old urban areas and urban expansion areas. Thus, Chinese cities aim for a fair allocation of waterbody during the growth process in statistics.

The overall changes in waterbody area over time for a large city clearly show that the concept of water protection must be strengthened, despite the waterbody loss per unit urban expansion suffered by all three city types decreasing since 2010 (Fig. 3). Moreover, the observed turning point in waterbody loss (ca. 2010) demonstrates that the protection of waterbodies and the environment in general has increased since the reckless era of economic development in China. Starting in the 1990s, the government has issued policies and regulations that strive to protect rivers, lakes, and wetlands. For example, in 1988, the Regulations on River Management of China prohibited the reclamation of land from lakes and rivers. In 2007, the “Blue Line” governance further emphasized waterbody protection within city limits, restricting landfills in waterbodies during urban development. Various provinces and cities also imposed special regulations for their regions. For example, Jiangsu province implemented the Lake Protection Regulations in 2004, which emphasized the protection of waterbodies. In 2006, Zhejiang Province promulgated measures for the management of waterbodies involved in construction projects, which initiated water protection in urban construction. Many regions then promulgated regulations to protect waterbodies from urban expansion. In addition, to construct an eco-friendly city in the Xiong’an New Area, the plan proposed 70% fraction of blue and green spaces and proposed strictly protecting waterbodies^40^. In 2017, the Ministry of Housing and Urban–Rural Development issued guidelines to strengthen ecological restoration of urban areas, stressing the concept of “sponge-city” construction and the ecological restoration of rivers, lakes, wetlands, and other waterbodies, and two ecological-restoration projects and urban restoration were undertaken to protect the natural urban water system^41^. To a certain extent, these measures reflect the change in attitudes towards waterbodies, gradually shifting from economically oriented rapid expansion to the joint development of urban economy and ecology, thereby enhancing the sustainability of the city.

**Figure 6:**
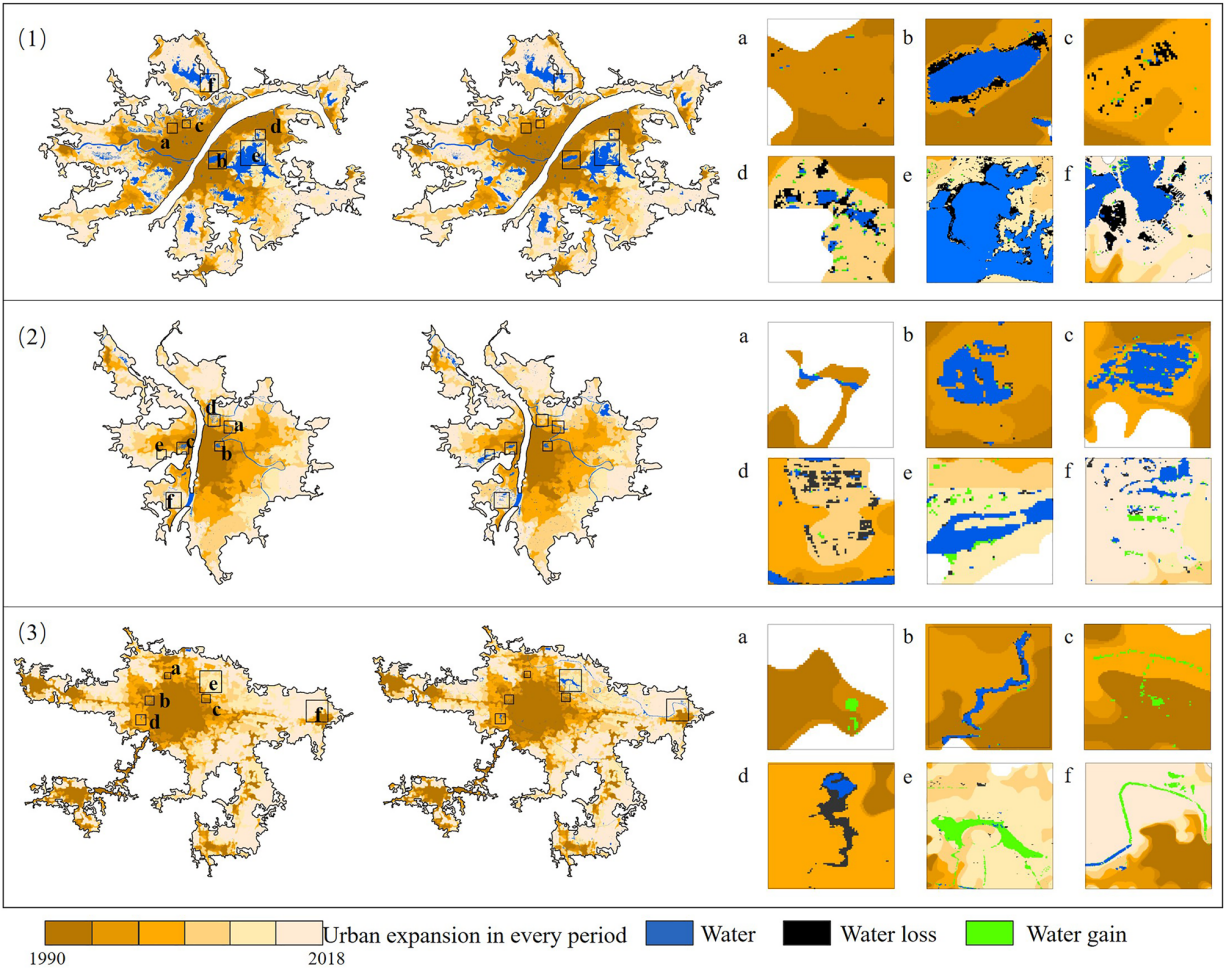
Variations in urban waterbodies of every period in typical cities: (1) Wuhan, (2) Changsha, and (3) Zhengzhou.

Cities will also face increasing uncertainties and risks, including climate change and natural disasters in future urbanization. Recently, serious urban waterlogging disasters related to the dramatic urban expansion in China have occurred with increasing frequency in many large- and medium-sized cities, causing incalculable losses to society. Although waterbodies generally occupy only a small part of a city, they sustain the urban ecological environment and regulate runoff within the urban area^42^. The hydrological cycle was disturbed and the permeable area decreased as a result of industrialization and urbanization. The sponge city program proposed to replace the gray infrastructure by using green infrastructure to retain more city's natural resource. This policy requires the maintenance of more natural elements in rapid urbanization and the creation of a porous and interconnected urban eco-spatial pattern through rivers, ponds, etc. And urban water systems may be managed more naturally by connecting existing buildings to green infrastructure^43^. It is thus urgent to focus more attention on coordinating urban development of construction land with ecological spaces. Waterbodies in Type-III cities have generally increased in scope during the study period (1990–2018; see Figs. 5 and 6). For example, Changsha constructed artificial lakes and created water landscapes to sustain the ecology of the city and provide new avenues of local tourism. People who proposed ecological cities believe that the inefficient use of resources degrades urban development^44^. The quantitative research presented herein reveals the shift in China’s development strategy, which is crucial to the future ecological development of cities. This strategy holds that urban planning must now consider the ecological function of waterbodies, which includes flood control, drainage, water storage, and biodiversity sustenance. In addition, the importance of urban blue and green infrastructure should be paramount, as dictated by science for the construction of sustainable cities^45^.

This analysis provides a valuable argument for protecting of surface waterbodies in urbanization; however, a challenge remains in evaluating encroached waterbodies because of the difficulty of eliminating the influence of natural conditions such as climate on waterbodies^46^. To negate this effect, we used the 3-year waterbody frequency index to describe surface waterbodies. Limited by the data on waterbodies with 30 m resolution, waterbodies obscured by trees are difficult to identify, making it difficult to detect all waterbodies.

## Methods

This study quantitatively assessed waterbody loss due to urban expansion of large Chinese cities. We first extracted multi-temporal urban boundaries to determine the expansion of cities of over one million in population from 1990 to 2018. The monthly surface-water dataset was then used to identify surface waterbodies in the study period. Depending on the ratio of surface waterbody area to urban area, cities were further divided into three categories (i.e. water-abundant, water-medium, water-deficient). Finally, we quantified the rate of waterbody loss and evaluated the spatial and temporal variation of waterbody loss as a function of urban expansion and according to city type.

### GUB dataset

The Global Urban Boundary (GUB) dataset (http://data.ess.tsinghua.edu.cn) was used to determine urban expansion. GUB provides data on built-up areas over 30 years, with a spatial resolution of 30 m. In the GUB dataset, nonurban areas (such as green space and water space) surrounded by artificial impervious areas are filled within the urban boundary and removed by the algorithm, which is consistent with global mapping methods. The continuous urban boundary was demarcated by morphological image processing methods, which have an overall accuracy of over 90%. In this dataset, extensive water and forests are excluded, and the impervious surface within the urban boundaries accounts for about 60% of the total surface area^47^. Compared with urban boundaries obtained from night-time light, GUB better separates urban areas from surrounding nonurban areas.

### Monthly waterbody dataset

We selected the JRC Monthly Water History V1.3 dataset(https://global-surface-water.appspot.com/), which is available from the Google Earth Engine, as the basis for representing surface waterbodies^48^. This data collection, which was produced by using images from the Landsat series, contains 442 images of global monthly waterbody area from March 1984 to December 2020. In this dataset, the validation confirmed that fewer than 1% of waterbodies were incorrectly detected, and fewer than 5% of waterbodies were missed altogether. We chose this dataset due to the long-term spatial distribution of waterbodies and due to mountain shadows and urban-constructions masking, which reflects the real changes in waterbodies.

### Theoretical background

It is well known that cities have high concentrations of population and resources and expand spatially during development. There are many different perspectives on the size of cities, and studies have mostly used urban density and population to characterize them. However, because it is challenging to standardize data sources and quality, there is no unified quantitative standard^49^. Urban construction has concentrated human activity and brought about changes in land types. Cities are also identified as physical spaces, which can be defined as the built environment^50,51^. The built environment, which includes structures like buildings, roads, and other artificial constructions, is sometimes referred to as a non-natural environment^52^.

Rural is the antithesis of urban. As large cities have spread outward in developing nations like Asia, a transitional fringe has been created by the gradual blurring of the line separating urban and rural areas^53^. According to McGee, good locations, easy access, and sizable agricultural land all contribute to the development potential of large cities. Thus, between urban and rural areas, there are transitional areas of active spatial morphological change known as desakota^33,54^. The peri-urban areas, like desakota, are gradually developed and incorporated into original built-up urban areas in urbanization. The original landscape, which included agricultural land, vegetation, and waterbodies, gradually changed into an urban land use type, i.e. impervious surface, and thus the city continues to expand outwards. Waterbody, an essential ecological element, has been heavily developed or filled in during urbanization, which may present dangerous ecological risks. In this paper, we identified the urban boundaries based on physical space to explore the encroachment activities on waterbodies during the urbanization of large cities. We determined whether existing waterbodies were transformed into urban waterbodies or encroached upon and whether waterbodies were increased in the expansion of urban boundaries, thus proposing strategies for protecting waterbodies in the future.

### Extracting the extent of large Chinese cities from GUB dataset

To characterize urban expansion, GUB data are selected as the original data for urban boundary selection. The Chinese administrative scale of municipalities is not exclusively urban, but also includes rural areas. In our study, cities were defined as municipal districts excluding the vast countryside within the administrative boundaries of prefecture-level cities. We identified urban areas based on the physical boundaries from the perspective of remote sensing, which can precisely track urban expansion^51^.

In this work, we selected 159 cities with a population of over one million in 2018 based on the average annual population of urban districts from the *2019 China City Statistical Yearbook* (Fig. S1). Taiwan, Hong Kong, and Macau are omitted. According to statistics, China had 160 cities with populations exceeding one million in 2018. However, due to the lack of data for the built-up area in 1990, Guang’an was not included in the study. We thus obtained 159 cities from the GUB dataset. Due to numerous fragmented patches within the administrative boundary, the population identified the main urban areas, and max patch areas were comprehensively based on the urban boundaries. Through manual detection and adjustment of the map, we determined that the location of the extracted urban area was consistent with that of the municipal government, and the boundary was extracted for each period. We took the growth area as the expansion area, with the original area being the city at the onset of each period (Fig. S3).

We used the average annual urban growth (AUG) rate to characterize the rate of urban expansion, as is widely done to evaluate urban expansion^55,56^. It is calculated as

$${\text{AUG}} = \left[ {\frac{{Land_{t1} }}{{Land_{t0} }}^{{\frac{1}{t1 - t0}}} - 1} \right] \times 100\% ,$$

where $Land_{t0}$ and $Land_{t1}$ represent the urban land area at time *t*_0_ and *t*_1_, where *t*_0_ and *t*_1_ are the start and end of the given study period.

### Identification of urban waterbodies

Urban waterbodies contain all the components of urban flow networks above the ground and include natural waterbodies such as lakes, rivers, streams, and wetlands and artificial waterbodies such as parks and ponds^48^. We identified all waterbodies existing within the urban boundary as urban waterbody. Considering urban expansion, urban waterbodies vary as urban boundary shift at different stages. Our study explored how the original waterbodies changed under urban expansion, including whether they were kept as urban waterbodies or encroached upon. Considering the dryness or wetness of each year, we used the data for 3 years (36 months) around each period (1990, 1995, 2000, 2005, 2010, 2015, and 2018) to describe the waterbody. Not all waterbodies could be detected for each month of the year; for example, freezing may prevent waterbodies from being detected. To cover seasonal and permanent waterbodies, we used the waterbody frequency index (WFI), which is calculated as the fraction of waterbody months within the 3 years to identify stable waterbodies pixel by pixel^57^. The spatial distribution of each waterbody was then mapped comprehensively for each period. By comparing the extracted waterbody with the long-time-series high-resolution remote-sensing images from Google Earth, we found that the extracted waterbodies fit the actual waterbody distribution quite well (Fig. S2):

$$WFI\left( i \right) = \frac{WM\left( i \right)}{{DM\left( i \right)}}$$

where WFI(i) is the water occurrence for pixel *i* in the images before and after the given year, and *i* is the pixel number for the study area. WM(*i*) is the number of months during which the waterbody is detected in *i* pixel over the 3 years. DM(*i*) is the number of months during which the data are available in pixel *i*. If the waterbody frequency index of a pixel is greater than 25%, this pixel is considered as a waterbody; otherwise, it is not.

### City classification based on surface waterbody

Cities with over one million in population may not be short of waterbodies, but significant differences remain in surface waterbody abundance. Due to large differences in city size, it is inappropriate to use waterbody area as a criterion. Considering the influence of urban expansion, we ranked 159 cities according to the indicator of waterbody fraction (WF), namely the fraction of the original surface water within the urban boundary in 2018. Waterbodies not impacted by urbanization were taken as the original surface waterbody, which used the average surface waterbody from 1985 to 1991 as baseline. We used the natural break method to divide cities into abundant, moderate, and deficient levels (referred to as Type I, Type II, and Type III, respectively) and evaluate the abundance of waterbodies in cities. Based on the waterbody fraction (WF) value, which is calculated as follows:

$${\text{WF}} = \frac{{Water_{origin} }}{{Land_{2018} }}$$

where WF is used to judge the urban waterbody abundance in cities. $Water_{1990}$ is the origin surface waterbody area (used the year in 1985–1991) in the urban boundary of 2018, $Land_{2018}$ the urban land area in the urban boundary of 2018.

### Temporal characteristic of waterbody loss and gain

To understand the spatial–temporal features of surface waterbodies, we used five normalized indicators to compare waterbody variations between cities during urban expansion from the overall perspective and from the city perspective.

The variation in original natural waterbodies reflects the intensity of the natural resource development in urban expansion. We summarized the reduction and preservation of original waterbodies in urban expansion areas with a population of over one million to represent the encroachment of urban expansion on waterbodies:

$$WL = \frac{{\sum NWL_{t0\_t1} \left( i \right)}}{{\sum W_{t0} \left( i \right)}} \times 100\%$$

$$WP = \frac{{\sum (W_{t0} \left( i \right) - NWL_{t0\_t1} \left( i \right))}}{{\sum W_{t0} \left( i \right)}} \times 100\%$$

where *i* labels the city within the 159 cities, WL and WP are the fractions of waterbody loss and preservation in urban expansion areas of all cities, $NWL_{t0\_t1}$ is the net waterbody loss during period *t*_0_–*t*_1_ $, and W_{t0}$ is the natural waterbody in the urban expansion area at time *t*_0_.

To estimate the net waterbody loss caused by urban expansion at various stages, we used the standardized indicator, annual average net waterbody loss rate (ANWL), to compare waterbody loss speeds over time. This indicator is independent of the difference in waterbody abundance and can be compared over time. Waterbody loss is one part of the impact of urbanization; the other is waterbody gain. We used the same method to evaluate the annual average net waterbody gain rate (ANWG). The formulas are

$$A{\text{NWL}} = \frac{{NWL_{t0\_t1} }}{{W_{t0} \left( {t1 - t0} \right)}} \times 100\%$$

$$ANWG = \frac{{NWG_{t0 - t1} }}{{W_{t0} \left( {t1 - t0} \right)}} \times 100\%$$

where NWL and NWG are the net waterbody loss and gain, respectively, and the other abbreviations are the same as above.

Considering the direct impact of urban expansion, we used a normalized indicator, the average net waterbody loss velocity of urban expansion ($AWLV$), which refers to the amount of waterbody encroachment per unit urban expansion area. It quantifies the time-heterogeneity of waterbody loss due to urban expansion and is calculated as follows:

$$AWLV = \frac{{NWL_{t0\_t1} }}{{Land_{t1} - Land_{t0} }}$$

We calculated these indicators for the six expansion periods (1990–1995, 1995–2000, 2000–2005, 2005–2010, 2010–2015, and 2015–2018) (Fig. 3). In the study, if the waterbody pixel count is zero at the onset of the period, the indicator for the period is abnormal and thus excluded.

## Supplementary Information

Supplementary Information.

## Data Availability

The datasets used and/or analysed during the current study available from the corresponding author on reasonable request.
