# Supplementary material for: Waterbody loss due to urban expansion of large Chinese cities in last three decades
Source: Sci Rep. 2022 Oct 19;12:17498. doi: 10.1038/s41598-022-22286-x (PMC9582205; doi:10.1038/s41598-022-22286-x)
Supplement: Supplementary file 1 — Supplementary Information. [file 41598_2022_22286_MOESM1_ESM.pdf]

Supplementary Material

Waterbody loss due to urban expansion of large Chinese cities in last three decades

Wu Xiao[1,2], Wenqi Chen[1,2], Wenze Yue*[1,2], Jingxuan Mu[1,2], Jianpeng Xu[1,2]
1 Department of Land Management, Zhejiang University, 310058, Hangzhou, China
2 Land Academy for National Development, Zhejiang University, Hangzhou, China

Table S1 Waterbody loss in every urban expansion period

City type		1990-1995	1995-2000	2000-2005	2005-2010	2010-2015	2015-2018
Type I	Waterbody loss area(km^2)	6.17	16.60	42.78	105.05	37.49	36.97
	Original waterbody area(km^2)	50.87	131.95	153.60	336.50	188.19	308.09
	The proportion of waterbody loss(%)	12.13	12.58	27.85	31.22	19.92	12.00
Type II	Waterbody loss area(km^2)	3.96	10.08	14.73	52.55	34.88	27.71
	Original waterbody area(km^2)	19.82	67.50	75.32	157.03	167.15	218.59
	The proportion of waterbody loss(%)	19.97	14.93	19.56	33.46	20.87	12.68
Type III	Waterbody loss area(km^2)	1.89	7.04	8.10	26.07	15.11	17.09
	Original waterbody area(km^2)	9.18	23.67	40.91	86.19	70.63	145.02
	The proportion of waterbody loss(%)	20.58	29.73	19.80	30.25	21.39	11.79

Table S2 Top 16 cities by urban growth area and rate

Rank	City	Growth area（km^2）	City	Growth rate
1	Beijing	3455.82	Putian	67.50
2	Shanghai	3409.59	Huzhou	40.48
3	Suzhou	2787.05	Guiyang	38.80
4	Guangzhou	2236.85	Taizhou	35.97
5	Foshan	2002.22	Jieyang	33.57
6	Dongguan	2000.64	Shaoxing	32.42
7	Wuxi	1741.23	Suzhou	31.65
8	Hangzhou	1727.95	Dongguan	28.18
9	Quanzhou	1629.95	Xiamen	26.92
10	Tianjin	1585.45	Qingyuan	25.51
11	Zhengzhou	1510.69	Huizhou	25.49
12	Weifang	1304.74	Nantong	22.69
13	Chengdu	1194.85	Ningbo	22.11
14	Wuhan	1192.48	Zhongshan	21.09
15	Shenzhen	1173.32	Wuxi	19.21
16	Linyi	1150.12	Nanchong	18.89

Table S3 Urban waterbody changes in typical cities (km^2)

		1990-1995	1995-2000	2000-2005	2005-2010	2010-2015	2015-2018	Summary
Wuhan	Gain	0.25	0.31	0.44	1.92	0.85	4.21	7.97
	Loss	0.08	0.69	1.03	6.42	6.95	5.00	20.17
	Preserve	0.29	1.96	3.34	2.73	2.60	11.77	22.70
	Change	0.17	-0.39	-0.59	-4.50	-6.10	-0.79	-12.20
Changsha	Gain	0.08	0.10	0.39	0.72	0.57	1.98	3.84
	Loss	0.00	0.12	0.65	1.74	0.46	1.01	3.98
	Preserve	0.29	1.96	3.34	2.73	2.60	11.77	22.70
	Change	0.08	-0.02	-0.26	-1.02	0.11	0.97	-0.14
Zhengzhou	Gain	0.11	0.06	0.35	0.34	4.49	6.96	12.30
	Loss	0.00	0.14	0.07	0.60	0.36	0.38	1.56
	Preserve	0.04	0.33	0.83	0.45	0.38	4.71	6.74
	Change	0.11	-0.08	0.28	-0.26	4.12	6.59	10.75

City classification results

Type I cities have abundant urban surface waterbody and occupy 52.4% of the waterbody in all cities. These 29 cities are mainly distributed in southern China, concentrated in the Pearl River Delta and along the Yangtze River. Type II cities, namely cities with water resources at a moderate level, are mostly distributed in the Yangtze River Delta, accounting for 51 cities. Type III represents surface waterbody deficiency, which has nearly half of the cities (79) but only accounts for 14.2% of the total waterbody. These cities are mainly distributed in the area north of the Yangtze River in China, concentrated in the North China Plain (Fig.S1).

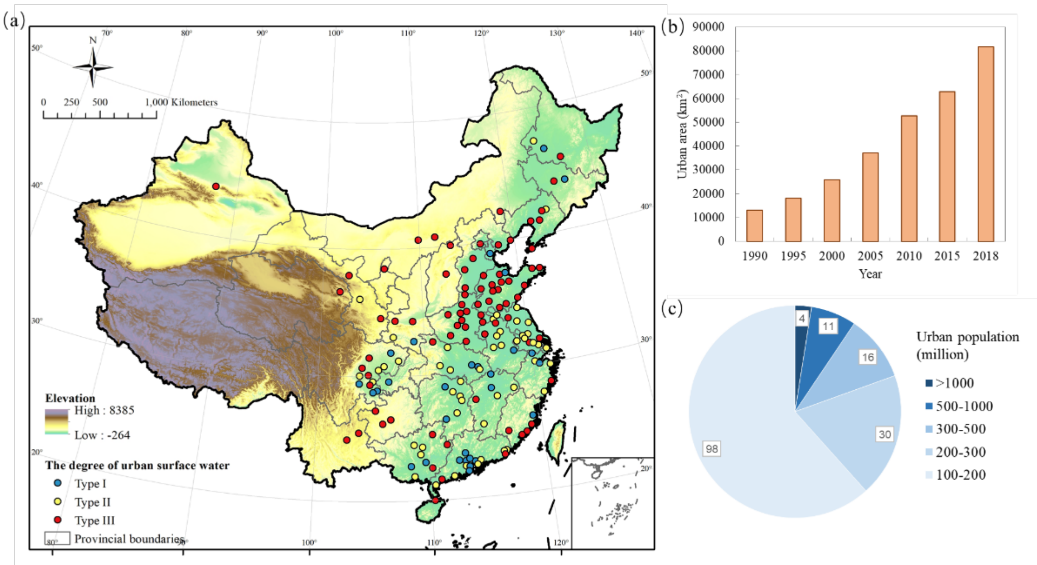

Fig.S1 Overview of the study area (a shows the cities distribution and the cities classification by waterbody abundance; b is the total change of urban area, and c is the number of cities with different levels of urban population). The map was generated by ESRI ArcGIS 10.2 software available at ESRI website (https://www.esri.com/en-us/arcgis/products/arcgis-platform/overview). And the administrative boundary shapefile is available at RESDC website (https://www.resdc.cn/).

Extracted waterbody validation

We took Beijing, Wuhan, Shanghai, Guangzhou as the sample to validate the accuracy of extracted waterbody. We randomly selected 50 sample points in the four regions, a total of 200. And we used visual interpretation to identify waterbodies and non-waterbodies sample points based on the Google image in 2018 for validation, and compare with our extracted results in 2018. The results show an overall accuracy of 89%, and it can effectively extract most urban waterbodies.

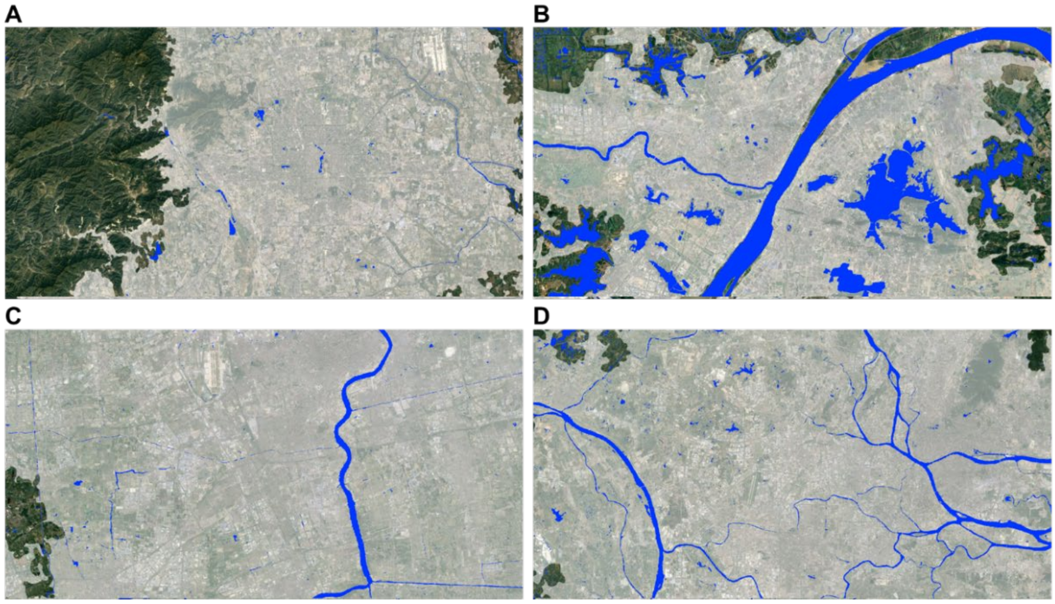

Fig.S2 Waterbody detection in the four case study areas：(A)Beijing, (B)Wuhan, (C)Shanghai, (D)Guangzhou. The source of background images comes from Google Earth.

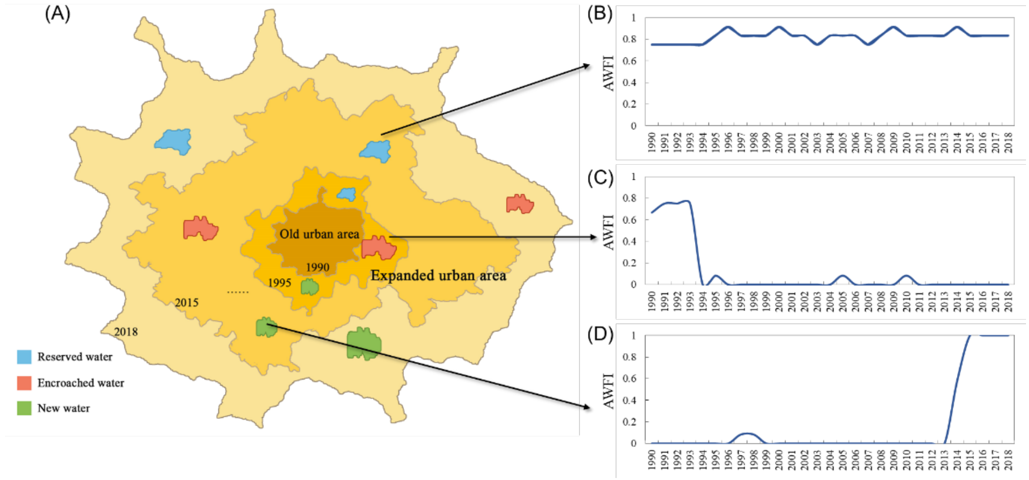

Fig.S3 Schematic diagram of waterbody change under urban expansion (a shows the waterbody variation in the space dimension, b-d shows annual waterbody frequency index (AWFI) curves for three kinds of waterbody change in the time dimension)

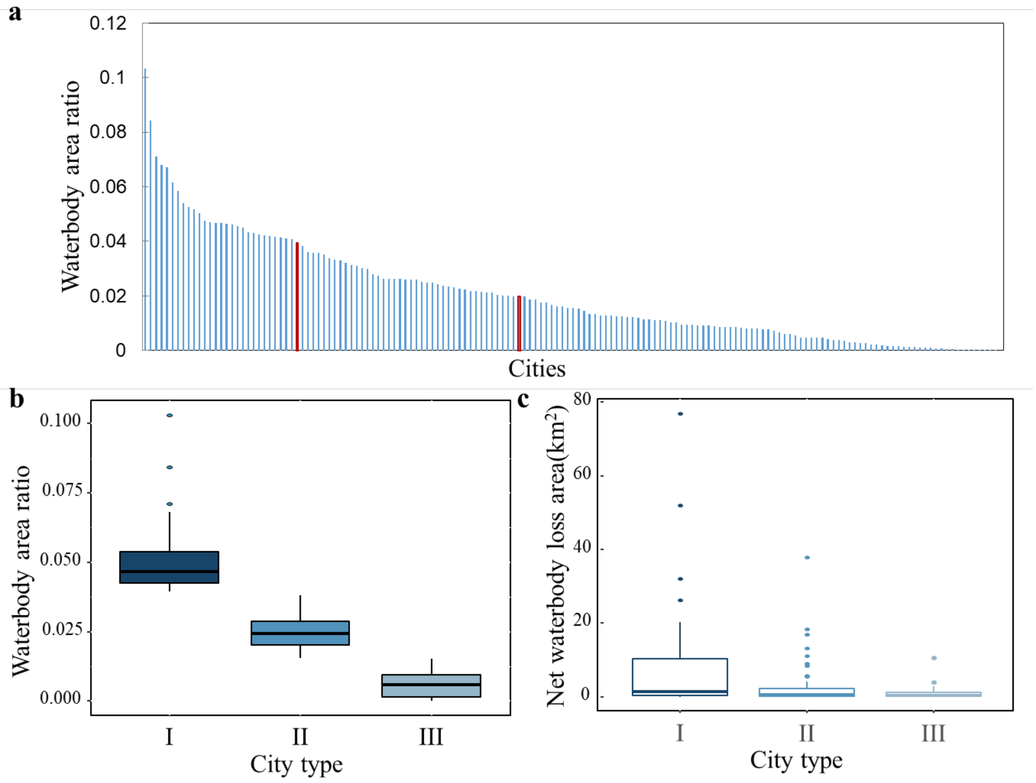

Fig.S4 The waterbody in three types of cities (a. the ratio of waterbody to the urban land for all cities; b. the ratio of waterbody area to urban land area among cities of different types; c. the amount of net waterbody loss among cities of different types). Cities were divided into three levels based on the waterbody proportion by Natural Breaks.
